# Can comprehensive background knowledge be incorporated into substitution models to improve phylogenetic analyses? A case study on major arthropod relationships

**DOI:** 10.1186/1471-2148-9-119

**Published:** 2009-05-27

**Authors:** Björn M von Reumont, Karen Meusemann, Nikolaus U Szucsich, Emiliano Dell'Ampio, Vivek Gowri-Shankar, Daniela Bartel, Sabrina Simon, Harald O Letsch, Roman R Stocsits, Yun-xia Luan, Johann Wolfgang Wägele, Günther Pass, Heike Hadrys, Bernhard Misof

**Affiliations:** 1 Molecular Lab, Zoologisches Forschungsmuseum A. Koenig, Bonn, Germany; 2 Department of Evolutionary Biology, University Vienna, Vienna, Austria; 3 ITZ, Ecology & Evolution, Stiftung Tieraerztliche Hochschule Hannover, Hannover, Germany; 4 Institute of Plant Physiology and Ecology, Shanghai Institutes for Biological Sciences, Chinese Academy of Sciences, Shanghai, PR China; 5 Department of Ecology and Evolutionary Biology, Yale University, New Haven, CT, USA; 6 UHH Biozentrum Grindel und Zoologisches Museum, University of Hamburg, Hamburg, Germany

## Abstract

**Background:**

Whenever different data sets arrive at conflicting phylogenetic hypotheses, only testable causal explanations of sources of errors in at least one of the data sets allow us to critically choose among the conflicting hypotheses of relationships. The large (28S) and small (18S) subunit rRNAs are among the most popular markers for studies of deep phylogenies. However, some nodes supported by this data are suspected of being artifacts caused by peculiarities of the evolution of these molecules. Arthropod phylogeny is an especially controversial subject dotted with conflicting hypotheses which are dependent on data set and method of reconstruction. We assume that phylogenetic analyses based on these genes can be improved further i) by enlarging the taxon sample and ii) employing more realistic models of sequence evolution incorporating non-stationary substitution processes and iii) considering covariation and pairing of sites in rRNA-genes.

**Results:**

We analyzed a large set of arthropod sequences, applied new tools for quality control of data prior to tree reconstruction, and increased the biological realism of substitution models. Although the split-decomposition network indicated a high noise content in the data set, our measures were able to both improve the analyses and give causal explanations for some incongruities mentioned from analyses of rRNA sequences. However, misleading effects did not completely disappear.

**Conclusion:**

Analyses of data sets that result in ambiguous phylogenetic hypotheses demand for methods, which do not only filter stochastic noise, but likewise allow to differentiate phylogenetic signal from systematic biases. Such methods can only rely on our findings regarding the evolution of the analyzed data. Analyses on independent data sets then are crucial to test the plausibility of the results. Our approach can easily be extended to genomic data, as well, whereby layers of quality assessment are set up applicable to phylogenetic reconstructions in general.

## Background

Most recent studies that focused on the reconstruction of ancient splits in animals, have relied on 18S and/or 28S rRNA sequences, e.g. [1]. These data sets strongly contributed to our knowledge of relationships, however, several nodes remain that are suspected of being artifacts caused by peculiar evolutionary rates which may be lineage specific. Particular unorthodox nodes were discussed as long branch artifacts, others were held to be clusters caused by non-stationary evolutionary processes as indicated by differences in nucleotide composition among the terminals. The reconstruction of ancient splits seems to be especially dependent on taxon sampling and character choice, since in single lineages the signal-to-noise ratio is consistently marginal in allowing a reasonable resolution. Thus, quality assessment of data via e.g. secondary structure guided alignments, discarding of randomly similar aligned positions or heterogeneity of the data set prior to analysis is a crucial step to obtain reliable results. Arthropod phylogeny is especially suitable as a case study, since their ancient and variable phylogenetic history, which may have included intermittent phases of fast radiation, impedes phylogenetic reconstruction.

### Major arthropod relationships

While currently there is wide agreement about the monophyly of Arthropoda, relationships among the four major subgroups (Chelicerata, Myriapoda, Crustacea, Hexapoda) remain contested, even the monophyly of each of the subgroups has come under question. The best supported relationship among these subgroups seems to be the clade comprising all crustaceans and hexapods. This clade, named Pancrustacea [2], or Tetraconata [3], is supported by most molecular analyses, e.g. [1,4-14]. Likewise, the clade has increasingly found support from morphological data [3,15-18], especially when malacostracans are directly compared with insects. Most of these studies reveal that crustaceans are paraphyletic with respect to a monophyletic Hexapoda. However, most analyses of mitochondrial genes question hexapod monophyly [19-22]. Additionally, various crustacean subgroups are discussed as potential hexapod sister groups. Fanenbruck et al. [15] favored a derivation of Hexapoda from a common ancestor with Malacostraca + Remipedia based on neuroanatomical data. In recent molecular studies, either Branchiopoda [12] or Copepoda [1,11,23] emerged as the sister group of Hexapoda. The Pancrustacea hypothesis implies that Atelocerata (Myriapoda + Hexapoda) is not monophyletic. In most of the above mentioned molecular studies, the Myriapoda appear at the base of the clade Mandibulata or as the sistergroup to Chelicerata. The combination of Chelicerata + Myriapoda [1,7,13,14,24] was coined Paradoxopoda [11] or Myriochelata [10]. It seems that this grouping can be partly explained by signal erosion [25], and likewise is dependent on outgroup choice [26]. In addition, the most recent morphological data is consistent with the monophyly of Mandibulata [27], but not of Myriochelata. Almost no morphological data corroborate Myriochelata except for a reported correspondence in neurogenesis [28]; this however alternatively may reflect the plesiomorphic state within Arthropoda [29,30]. Within Hexapoda, relationships among insect orders are far from being resolved [31-35]. Open questions concern the earliest splits within Hexapoda, e.g. the monophyly or paraphyly of Entognatha (Protura + Diplura + Collembola) [9,19,22,32,34,36-45].

### Goals and methodological background

The aim of the present study is to optimize the phylogenetic signal contained in 18S and 28S rRNA sequences for the reconstruction of relationships among the major arthropod lineages. A total of 148 arthropod taxa representing all major arthropod clades including onychophorans and tardigrades (the latter as outgroup taxa) were sampled to minimize long-branch artifacts [25]. A new alignment procedure that takes secondary structure into account is meant to corroborate the underlying hypotheses of positional homology as accurately as possible. A new tool for quality control optimizes the signal-to-noise ratio for the final analyses. In the final step, we try to improve the analyses by fitting biologically realistic mixed DNA/RNA substitution models to the rRNA data. Time-heterogeneous runs were performed to allow for lineage specific variation of the model of evolution.

The use of secondary structure information both corroborates hypotheses of positional homology in the course of sequence alignment, as well as helps to avoid misleading effects of character dependence due to covariation among sites. It was demonstrated that ignoring correlated variance may mislead tree reconstructions biased by an overemphasis of changes in paired sites [34,46,47]. Evolutionary constraints on rRNA molecules are well known, for example constraints resulting from secondary structure interactions. The accuracy of rRNA comparative structure models [48-50] has been confirmed by crystallographic analyses [51,52]. Based on this background knowledge, rRNA sequences are an ideal test case to study the effect of biologically realistic substitution models on tree reconstructions.

Recent studies of genome scale data revealed that a careful choice of biologically realistic substitution models and model fitting are of particular importance in phylogenetic reconstructions [53-55]. The extent, however, to which biological processes can/should be modeled in detail is still unclear. The analyses of rRNA sequences can still deliver new insights in this direction, since the relatively comprehensive background knowledge allows to better separate different aspects of the substitution processes. In order to model covariation in rRNA sequences, we estimated secondary structure interactions by applying a new approach implemented in the software RNAsalsa [56] (download available from ), which helps to accommodate inadequate modeling (e.g. missing covariotide effects) of rRNA substitution processes in deep phylogenetic inference [34,57]. Essentially, this approach combines prior knowledge of conserved site interactions modeled in a canonical eukaryote secondary structure consensus model with the estimation of alternative and/or additional site interactions supported by the specific data. Inferred site covariation patterns were used then to guide the application of mixed substitution models in subsequent phylogenetic analyses.

Finally, we accounted for inhomogeneous base composition across taxa, a frequently observed phenomenon indicating non-stationary substitution processes [58-60]. Non-stationary processes, if present, clearly violate assumptions of stationarity regularly assumed in phylogenetic analyses [60-62]. Thus, we modeled non-stationary processes combined with the application of mixed DNA/RNA substitution models in a Bayesian approach using the *PHASE-2.0 *software package [63] to provide a better fit to our data than standard substitution models [60,64]. In *PHASE-2.0 *a nonhomogeneous substitution model is implemented [...] "by introducing a reversible jump Markov chain Monte Carlo method for efficient Bayesian inference of the model order along with other phylogenetic parameters of interest" [60].

Application of a new hierarchical prior leads to more reasonable results when only a small number of lineages share a particular substitution process. Additionally *PHASE-2.0 *includes specialized substitution models for RNA genes with conserved secondary structure [60].

## Results

**Figure 1:**
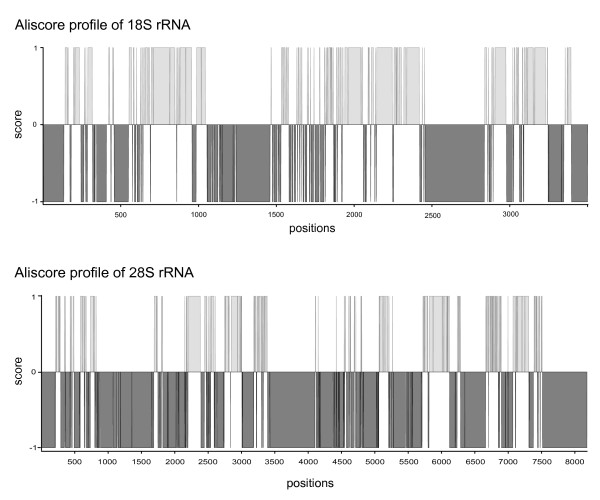
**ALISCORE consensus profiles of rRNA alignments**. **1A **ALISCORE consensus profile of the 18S rRNA alignment generated from single profiles of aligned positions after applying the sliding window approach based on MC resampling. Randomly similar sections (1873 positions) show negative score values or positive values non-random similarity (y-axis). Sequence length and positions are given on the x-axis. **1B **ALISCORE consensus profile of the 28S rRNA alignment generated from single profiles of aligned positions after applying the sliding window approach based on MC resampling. Randomly similar sections (5712 positions) show negative score values or positive values for non-random similarity (y-axis). Sequence length and positions are given on the x-axis.

We contributed 103 new and nearly complete 18S or 28S rRNA sequences and analyzed sequences for 148 taxa (Additional file 1), of which 145 are Arthropoda *sensu stricto*, two onychophorans and *Milnesium *sp. (Tardigrada). The alignment of the 18S rRNA sequences comprised 3503 positions, and the 28S rRNA alignment 8184. The final secondary consensus structures included 794 paired positions in the 18S and 1326 paired positions in the 28S. The consensus structures contained all paired sites that in 60% or more sequences were detected after folding (default *s*3 = 0.6 in RNAsalsa). ALISCORE[65] scored 1873 positions as randomly similar (negative scoring values in the consensus profile) to the 18S and 5712 positions of the 28S alignment (Figure 1).

### Alignment filtering and concatenation of data

After the exclusion of randomly similar sections identified by ALISCORE, 1630 (originally 3503) of the 18S rRNA and 2472 (originally 8184) positions of the 28S rRNA remained. Filtered alignments were concatenated and used for analyses in *PHASE-2.0*. The concatenated alignment comprised 4102 positions.

### Split supporting patterns

**Figure 2:**
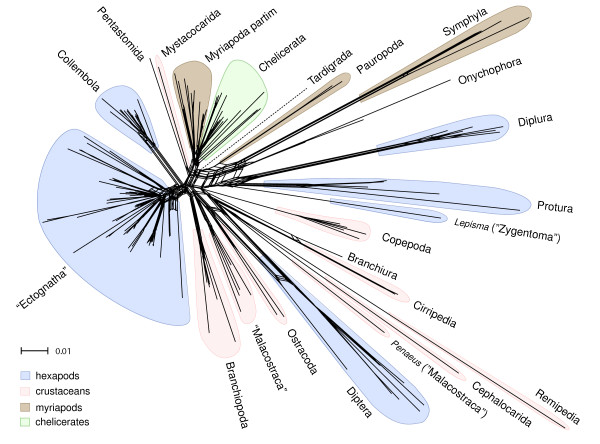
**Neighbornet graph of the concatenated 18S and 28S rRNA alignment**. Neighbornet graph based on uncorrected p-distances constructed in SplitsTree4 using the concatenated 18S and 28S rRNA alignment after exclusion of randomly similar sections evaluated with ALISCORE. Hexapods are colored blue, crustaceans red, myriapods brown and chelicerates green. Quotation marks indicate that monophyly is not supported in the given neighbornet graph.

The neighbornet graph, which results from a split decomposition based on uncorrected p-distances (Figure 2) and LogDet correction plus invariant sites model (see Additional file 2) pictured a dense network, which hardly resembles a tree-like topology. This indicates the presence of some problems typical in studies of deep phylogeny: a) Some taxa like Diptera (which do not cluster with ectognathous insects), Diplura, Protura and Collembola each appear in a different part of the network with Diplura and Protura seperated from other hexapods, *Lepisma saccharina *(clearly separated from the second zygentoman *Ctenolepisma *that is nested within Ectognatha), Symphyla, Pauropoda, as well as Remipedia and Cephalocarida have very long branches. Consequently the taxa may be misplaced due to signal erosion or occurrence of homoplasies, and their placement in trees must be discussed critically [25]. The usage of the LogDet distance adjusts the length of some branches but does not decrease the amount of conflicts in deep divergence splits. b) The inner part of the network shows little treeness, which indicates a high degree of conflicting signal.

A remarkable observation seen in both phylogenetic networks is that some taxa have long stem-lineages, which means that the species share distinct nucleotide patterns not present in other taxa. Such well separated groups are Copepoda, Branchiopoda, Cirripedia, Symphyla, Collembola, Diplura, Protura and Diptera, while e.g. Myriapoda partim, Chelicerata and the Ectognatha (bristletails, silverfish/firebrats and pterygote insects) excluding Diptera share weaker patterns.

### Compositional heterogeneity of base frequency

We excluded in *PAUP *4.0b10 [66] parsimony uninformative positions explicitely for the base compositional heterogeneity test. Randomly similar alignment blocks identified by ALISCORE were excluded for both, the base compositional heterogeneity test and phylogenetic recontructions. 901 characters of the 18S rRNA and 1152 characters of the 28S rRNA were separately checked for inhomogeneous base frequencies. Results led to a rejection of the null hypothesis (*H*_0_), which assumes homogeneous base composition among taxa (18S: *χ*^2 ^= 1168.94, *df *= 441, *P *= 0.00; 28S: *χ*^2 ^= 1279.98, *df *= 441, *P *= 0.00). Thus, base frequencies significantly differed across taxa in both 18S and 28S data sets.

A data partition into stems and loops revealed 477 unpaired positions and 424 paired positions in the 18S, and 515 unpaired and 637 paired positions in the 28S. Separate analyses of all four partitions confirmed heterogeneity of base frequencies across taxa in all sets (*P *= 0.00 in all four partitions).

We repeated the homogeneity test for partitions as used in tree reconstruction, if base pairs were disrupted by the identification of the corresponding partner as randomly similar (ALISCORE), remaining formerly paired positions were treated as unpaired. Hence, 1848 characters of the concatenated alignment (18S: 706; 28S: 1142) were treated as paired in all analyses. Again the test revealed heterogeneity in unpaired characters of both the 18S and 28S (*P *= 0.00 for both genes; 18S: 506 characters; 28S: 567 characters). Examination at paired positions also rejected the null hypothesis *H*_0 _(18S, 395 characters included: *P *< 0.0003, 28S, 585 characters included: *P *= 0.00). Since non-stationary processes in all tests were strongly indicated, we chose to apply time-heterogeneous models to account for lineage-specific substitution patterns. To fix the number of "free base frequency sub-models" in time-heterogeneous analyses, we identified the minimal exclusive set of sequence groups. Based on *χ*^2^-tests the dataset could be divided into three groups for both rRNA genes. In both genes Diptera are characterized by a high A/T content and Diplura by a low A/T content. Exclusion of only one of the groups was not sufficient to retain a homogeneous data set (18S: excluding Diptera: *χ*^2 ^= 972.91, *df *= 423, *P *= 0.00, excluding Diplura: *χ*^2 ^= 532.13, *df *= 423, *P *< 0.0003; 28S: excluding Diptera: *χ*^2 ^= 986.72, *df *= 423, *P *= 0.00, excluding Diplura: *χ*^2 ^= 813.8, *df *= 423, *P *= 0.00). Simultaneous exclusion of both groups led to acceptance of *H*_0 _for 18S sequences (*χ*^2 ^= 342.22, *df *= 405, *P *= 0.99). For the 28S, after exclusion of both groups, *H*_0 _was still rejected (*χ*^2 ^= 524.98, *df *= 405, *P *< 0.0001). After sorting taxa according to base frequencies in ascending order, additional exclusion of *Peripatus *sp. and *Sinentomon erythranum *resulted in a homogeneous base composition for the 28S gene (*H*_0_: *χ*^2 ^= 434.99, *df *= 399, *P *= 0.1), likewise indicating that three sub-models are suffucient to cover the taxon set. We repeated the homogeneity-test for stem and loop regions of each gene seperately. The exclusion of Diplura was sufficient to obtain homogeneity in the loop regions for both genes (18S: 474 characters, *P *= 0.9757; 28S: 541 characters, *P *= 0.0684). For stem regions in the 18S it likewise was sufficient to exclude either Diptera (378 characters, *P *= 0.6635) or Diplura (385 characters, *P *= 0.99). These partitions would make two sub-models sufficient to cover the data set. However, in the stem regions of the 28S homogeneity was received only after the exclusion of both Diptera and Diplura (547 characters, *P *= 0.99). Since *PHASE-2.0 *does not allow to vary the number of chosen sub-models among partitions, we applied and fitted three sub-models to each data partition.

### Phylogenetic reconstructions

**Figure 3:**
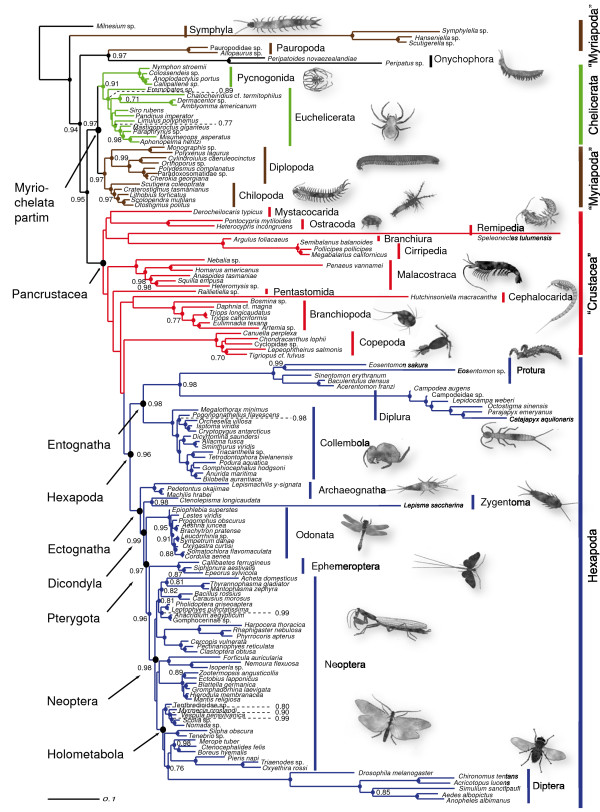
**Time-heterogeneous consensus tree**. Consensus tree from 56,000 sampled trees of the time-heterogeneous substitution process inferred by *PHASE-2.0*, graphically processed with Adobe Illustrator CS2. Support values below 0.70 are not shown (nodes without dots), nodes with a maximum posterior probability (pP) of 1.0 are represented by dots only. Quotation marks indicate that monophyly is not supported in the given tree.

**Figure 4:**
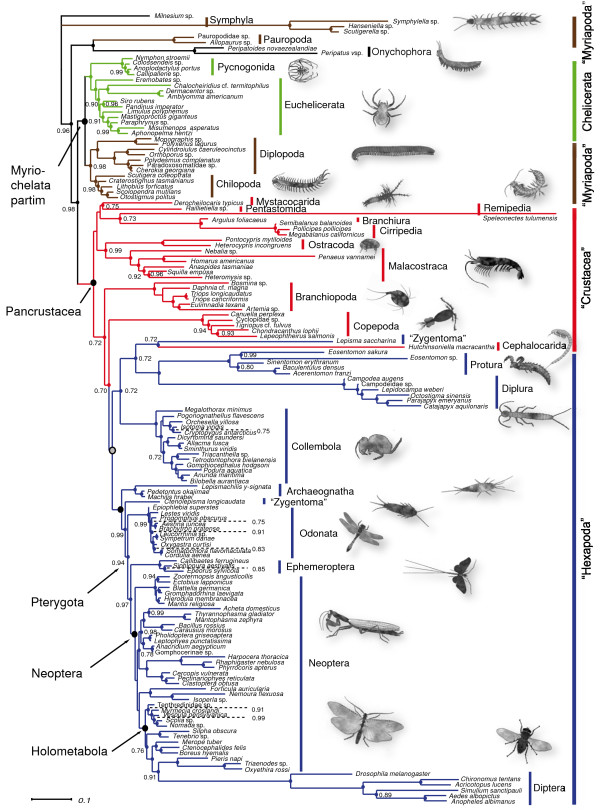
**Time-homogeneous consensus tree**. Consensus tree from 18,000 sampled trees of the time-homogeneous substitution process inferred by *PHASE-2.0*, graphically processed with Adobe Illustrator CS2. Support values below 0.70 are not shown (nodes without dots), nodes with a maximum posterior probability (pP) of 1.0 are represented by dots only. The grey dot indicates the clade containing all hexapod taxa including *Hutchinsoniella *(Crustacea) + *Lepisma *(Zygentoma); its node value is pP 0.58. Quotation marks indicate that monophyly is not supported in the given tree.

Three combinations of mixed DNA/RNA models (REV + Γ & RNA16I + Γ, TN93 + Γ & RNA16J + Γ and HKY85 + Γ & RNA16K + Γ) were compared to select the best model set. Overall model *ln *likelihoods converged for all tested mixed models after a burn-in of 250,499 generations in an initial pre-run of 500,000 generations. However, most parameters did not converge for the combined REV + Γ & RNA16I + Γ models, consequently, this set up was excluded from further analyses. For each of the remaining two sets a chain was initiated for 3 million generations, with a burn-in set to 299,999 generations. The applied Bayes Factor Test [[67,68], BFT], favored the TN93 + Γ & RNA16J + Γ model combination (2*lnB*_10 _= 425.39, harmonic mean *lnL*_0_(TN93 + Γ & RNA16J + Γ) = 79791.08; harmonic mean *lnL*_1_(HKY85 + Γ & RNA16K + Γ) = 80003.78). For each approach (Aditional file 3) all chains which passed a threshold value in a BFT were assembled to a metachain. Each resulting extended majority rule consensus tree was rooted with *Milnesium*. Node support values for clades were deduced from 56,000 sampled trees for the time-heterogeneous set (Figure 3) and from 18,000 sampled trees for the time-homogeneous set (Figure 4), detailed support values are shown in Additional file 3. Harmonic means of the *ln *likelihoods of included time-heterogeneous chains were compared against all *ln *likelihoods of included time-homogeneous chains (burn-in discarded) in a final BFT: the time-heterogeneous model was strongly favored (2*lnB*_10 _= 1362.13).

### Resulting topologies

Representatives of Symphyla and Pauropoda, already identified in the neighbornet graph as taxa with conspicuously long branches (Figure 2), assumed unorthodox positions in both trees which are clearly incongruent with morphological evidence and results obtained from other genes. Symphyla formed the sister group of all remaining arthropod clades, and Pauropoda clustered with Onychophora. Consequently, myriapods always appeared polyphyletic in both analyses. We consider these results as highly unlikely, since they contradict all independent evidence from morphology, development, and partly from other genes. In the following, we focus on major clades and point out differences between time-heterogeneous tree (Figure 3) and time-homogeneous tree (Figure 4) without considering the position of Symphyla and Pauropoda. Possible causes for the misplacement of these groups, however, will be treated in the discussion. Both analyses supported a monophyletic Chelicerata (pP 0.91 in the time-heterogeneous tree and maximal support in the time-homogeneous tree) with Pycnogonida (sea spiders) as sister group to remaining chelicerates. Pycnogonida received maximal support in both analyses. Euchelicerata received highest support in the time-homogeneous approach while this clade in the time-heterogeneous approach received a support of only pP 0.89. *Limulus polyphemus *(horseshoe crab) clustered within arachnids, but some internal relationships within Euchelicerata received only low support. Chilopoda always formed the sister group of a monophyletic Diplopoda in both analyses with high support. Within the latter the most ancient split lied between Penicillata and Helminthomorpha. This myriapod assemblage – Myriapoda partim – formed the sister group of Chelicerata, thus giving support to the Myriochelata hypothesis, respectively Myriochelata partim, when the long-branch clades Symphyla and Pauropoda are disregarded.

Pancrustacea showed always maximal support. The monophyly of Malacostraca and Branchiopoda received highest support in both approaches while their position varied. Branchiopoda was the sister group of the clade consisting of Copepoda + Hexapoda in the homogeneous tree (Figure 4), however the cephalocarid *Hutchinsoniella *nested within hexapods. Among hexapods, monophyly was unambiguously supported for Protura, Diplura, Collembola, Archaeognatha, Odonata, Ephemeroptera, Phasmatodea, Mantophasmatodea, Mantodea, Plecoptera, Hemiptera, Coleoptera, Hymenoptera, Lepidoptera and Diptera. Diplura clustered with Protura, and gave support to a monophyletic Nonoculata. Pterygota occurred in both topologies, well supported in the non-stationary tree (pP 0.97) and with moderate support (pP 0.94) in stationary tree. Within the winged insects, both analyses resolved Odonata as the sister group to a well supported monophyletic clade Ephemeroptera + Neoptera (heterogeneous: pP 0.96; homogeneous: pP 0.97), known as the "Chiastomyaria" clade [32,34,35,69]. Blattodea were always paraphyletic with respect to the isopteran representative. This assemblage formed a sister group relationship with Mantodea, thus giving support to a monophyletic Blattopteroidea or Dictyoptera while the position of Dictyoptera among hemimetabolan insects differed. Dermaptera always clustered with Plecoptera. Hemiptera (Heteroptera + Homoptera) in both approaches formed a clade with the remaining orthopterans + ((*Acheta *+ Mantophamsmatodea)Phasmatodea) with low statistical support. Caused by *Acheta *orthopteran insects appeared always polyphyletic. Within the monophyletic Holometabola (pP 1.0), Hymenoptera formed the sister group of the remaining taxa.

While the time-heterogeneous and time-homogeneous trees corresponded in overall topologies, they differed in a number of remarkable details.

1) Hexapoda, Entognatha, Ectognatha and Dicondylia were only reconstructed in the time-heterogeneous approach. 2) The cephalocarid *Hutchinsoniella *clustered among crustaceans as sister group to the Branchiopoda only in the heterogeneous approach, this clade formed the sister group to (Copepoda + Hexapoda) although with low support. 3) The time-homogeneous runs revealed highly supported (Malacostraca + Ostracoda) as the sister group to a clade ((Mystacocarida + Pentastomida) + (Branchiura + Cirripedia)). In contrast, in the time-heterogeneous analysis more terminal positioned Malacostraca are the sister group of a clade (Pentastomida((Cephalocarida + Branchiopoda) + (Copepoda + Hexapoda))). The altered postition of Pentastomida was only low supported in this tree. 4) In the homogeneous tree *Hutchinsoniella *emerged as sister taxon to *Lepisma *with low support (pP 0.72), and this cluster was positioned within the remaining hexapods (Figure 4). Hexapoda were monophyletic only in the time-heterogeneous approach, well supported (pP 0.96, Figure 3), with Copepoda as sister group, latter with low support (pP 0.69). 5) In the time-homogeneous tree (Figure 4), Copepoda emerged as sister group, again with a low support value (pP 0.70) of ((*Lepisma *+ *Hutchinsoniella*) + "Hexapoda"). 6) Entognatha (pP 0.98), and Ectognatha (pP 1.0) and Dicondylia (pP 0.99) were monophyletic only in the time-heterogeneous tree. 7) The time-heterogeneous tree showed the expected paraphyly of primarily wing-less insects with Archaeognatha as sister group to Zygentoma + Pterygota. 8) Within pterygote insects (Dermaptera + Plecoptera) emerged as sister group of Dictyoptera in the non-stationary tree, contrary as sister group of Holometabola in the stationary tree, both scenarios with negligible support.

## Discussion

Among arthropods 18S and 28S rRNA genes have the densest coverage of known sequences. Apart of some exceptions most studies on phylogenetic relationships at least partly rely on rRNA data. Often, however, only one of the genes was used, sometimes even just fragments of a gene [23,32,34,40,42,44,70-72], while only few studies used nearly complete 18S and 28S rRNA sequences [1,11,73]. Despite this wide usage, the reliability of reconstructions based on rRNA markers is still debated (for contradicting views see [34,74,75]. A major cause of concern is the pronounced site heterogeneity of evolutionary rates, the non-stationarity of base composition among taxa and rate variation in time. All three phenomena quickly lead to the erosion of phylogenetic signal [76]. On the one hand, our understanding of the molecular structure of other markers and about taxon-dependent processes of molecular evolution remains poor. On the other hand, our vast background knowledge regarding rRNA molecules offers a unique opportunity to study the effects of selection and application of substitution models in greater detail.

### Quality check and character choice in alignments

Phylogenetic signal in sequence data can get noisy due to (i) multiple substitution processes (saturation) and (ii) erroneous homology hypotheses caused by ambiguous sequence alignment. Both effects correspond in that they result in random similarity of alignment regions. Such noisy sections potentially bias tree reconstructions in several ways which have been appreciated for years but only recently been applied, that allow to account for these problems [25,54,77,78]. Exclusion of these ambiguously aligned or saturated regions can help to reduce noise, see e.g. [65]. If this topic is addressed at all, the majority of studies include a manual alignment check for untrustworthy regions [1,4,22,32,34,39,44,71-73]. Only some recent publications addressing arthropod relationships have used automated tools, e.g. [14,79,80].

To identify alignment sections of random similarity prior to tree reconstructions, we used ALISCORE, which, compared to the commonly used Gblocks [81], is not dependent on the specification of an arbitrary threshold [65]. To improve the signal-to-noise ratio we restricted our character choice to alignment sections which contained nucleotide patterns that differ from randomized patterns.

### Phylogenetic reconstruction methods

Arthropod phylogenies have been inferred with reconstruction methods like Maximum Parsimony, Maximum Likelihood and Bayesian approaches. We tried to implement knowledge about the evolution of rRNA in two ways: (i) the use of mixed DNA/RNA models is meant to account for known instances of character dependence due to compensatory mutations in stem regions, (ii) the application of time-heterogeneous models accounts for non-stationary processes that occurred in arthropod lineages. The consensus secondary structure of our dataset, generated with RNAsalsa, can be understood as a model parameter that defines site interactions and thus character dependence due to compensatory mutations [34,82,83]. Neglect of character dependence surely results in unrealistic support values. In single low supported nodes, where the signal-to-noise ratio is at the edge of resolution, such a neglect theoretically can even turn the balance between two competing hypotheses. Additionally a consensus secondary structure is necessary to apply a mixed model approach, since it determines whether the evolution of a given site is modeled by the DNA-model, or as part of a base-pair by the RNA-model. Within the mixed model approach, we opted for DNA-corresponding 16-state RNA models [63]. It can certainly be argued that the choice of 16-state models is problematic because it is difficult to fit these models to real data due to their parameter richness and heavy computational costs. However, even the best choice of a consensus secondary structure can only capture the predominantly conserved structural features among the sequences. This implies that the applied RNA models must be able to cope with mismatches in base-pairing. Less complex RNA models like those of the 6 and 7-state families either ignore mismatches completely or pool these mismatches into a single character state which produces artificial synapomorphies. Additionally, according to Schöninger and v. Haeseler [84], it is more likely that co-variation is a multiple step process which allows for the intermediate existence of instable (non Watson-Crick) pairs. These intermediate states are only described in 16-state RNA models.

Concerning rRNA-genes of arthropods, shifts in base composition are mentioned for Diptera, Diplura, Protura and Symphyla [1,23,34,44,73,85]. Since base compositional heterogeneity within a dataset can mislead phylogenetic reconstruction [61,86,87] and [60], some of these studies discussed observed but not incorporated non-stationary processes as possible explanations for misplacements of some taxa [11,23,24,44,73]. The selective exclusion of these taxa to test for misleading effects on the remaining topology, however, is not appropriate to test whether non-stationarity really fits as the causal explanation of the placement incongruent with other analyses. LogDet methods have been applied to compensate for variations of base frequencies [1,11,44], which leads to some independence of non-stationarity, but among site rate variation (ASRV) cannot be handled efficiently. After detecting compositional base frequency heterogeneity in our data, we chose a non-stationary approach implemented in *PHASE-2.0*. Because no previous study of arthropod phylogeny has used a time-heterogeneous approach including mixed DNA/RNA models, we compared this approach with a "classical" time-homogeneous setup. Our results prove that the time-heterogeneous approach produces improved likelihood values with improved branch lengths estimates and more realistic, though not perfect (see below), topology estimates. Since modeling of general time-heterogeneous processes is in its infancy and since its behavioural effect on real data is relatively unknown [60,61], we favored a set up accounting for the three different "submodels" corresponding to three base frequency categories in our dataset (Additional file 4). The application of the three submodels to individual branches in a tree by the MCMC process was not further constrained. This scheme allowed for a maximum of flexibility without losing the proper mix of parameters.

### Conflicting phylogenetic hypotheses and non-stationary processes of rRNA evolution

The comparison of our time-homogeneous approach to our time-heterogeneous one was not only meant to show improvements in the application of more realistic models, but also to indicate which incongruities of analyses of rRNA genes may be causally explained by non-stationary processes during the evolution of these genes.

In our time-homogeneous approach, the crustacean *Hutchinsoniella *(Cephalocarida) clustered with *Lepisma *(Zygentoma, Hexapoda) within enthognathans as sister group to Nonoculata (Protura + Diplura), (see Figure 4). This led to the polyphyly or paraphyly of several major groups (e.g. Hexapoda, Entognatha, Ectognatha, Dicondylia). In our time-heterogeneous analysis, Cephalocarida clustered as sister group to Branchiopoda. This result, although marginal supported, is congruent, at least, with some morphological data [88]. Most recent molecular studies have not included Cephalocarida, e.g. [1,11]. Regier et al. [12] reconstructed a sister group relationship of Remipedia and Cephalocarida (likewise represented by *Hutchinsoniella*), but his result also received only moderate bootstrap support. The same clade was presented in Giribet et al. [9] based on morphological and molecular data.

Independent of the sister group relationship of Cephalocarida within crustaceans, the correction of the misplacement of *Hutchinsoniella*, by allowing for non-stationary processes, has a major effect on the heuristic value of our analyses. Not only is the monophyletic status of Hexapoda, Entognatha, Ectognatha, Dicondylia supported after the correction, but likewise a causal explanation is given for the misplacement in the time-homogeneous approach, which cannot be accomplished by alternatively excluding the taxon. Our time-heterogeneous analyses resulted in a sister group relationship of Diplura and Protura, which lends support to a monophyletic Nonoculata within a monophyletic Entognatha. This result is congruent with trees published by Kjer [32], Luan et al. [44], Mallat and Giribet [1], and Dell'Ampio et al. [23]. Following Luan et al. [44] Dell'Ampio et al. [23] cautioned that Nonoculata may be an artificial cluster caused by a shared nucleotide bias and long branch attraction. Since this node is recovered with high support by our non-stationary approach, Nonoculata cannot be suspected of being an artificial group based on shared compositional biases alone. However, one must keep in mind that Protura and Diplura have longer branches than Ectognatha and Collembola (Figure 3 and 4), and long-branch effects may still be present. Thus monophyly of a clade Nonoculata still awaits support from a data set independent from rRNA sequences.

### Clades not affected by non-stationary processes

#### Symphyla and Pauropoda

Although we tried to break down long branches by a dense taxon sampling, some long-branch problems persisted. We cannot clearly address the reason but, due to the symptoms, assume that saturation by multiple substitution caused signal erosion (class II effect, [25]). To evaluate the impact on the topology of the very likely incorrect positions of Symphyla and Pauropoda, we repeated the time-heterogeneous analysis using a reduced dataset excluding these taxa. We limited the analysis to ten chains with 7, 000, 000 generations each (2, 000, 000 burn-in). Differences occurring in the inferred consensus topology (not shown) of the final three chains (15, 000, 000 generations) show that some nodes are still sensitive to taxon sampling, since e.g. Pycnogonida clustered with (Chilopoda + Diplopoda) after exclusion of pauropod and symphylan sequences. Also the crustacean topology changed, remaining long branch taxa *Hutchinsoniella *and *Speleonectes *clustered together in the reduced dataset, forming a clade with (Branchiura + Cirripedia).

#### Mandibulata versus Myriochelata

Analyses of rRNA sequences up till now were held to favor Myriochelata (Myriapoda + Chelicerata) over Mandibulata [1,4,11]. Our analyses provide no final conclusion with respect to this conflict, since the position of Pauropoda and Symphyla is unusual, it results in polyphyletic myriapods. The exact reconstruction of the position of myriapods within the Euarthropoda thus demands e.g. the application of new markers and suitable phylogenetic strategies.

#### Phylogenetic position of Malacostraca and Pentastomida

The position of Malacostraca differs among molecular studies. Often, Malacostraca emerge as nested within the remaining crustacean groups, e.g. [5,89]. Complete mitochondrial genomes place Malacostraca close to insects [90,91]. However, studies of rRNA sequences recover this group as the sister group to all remaining crustaceans [1,11,92]. Since in our stationary tree monophyletic Malacostraca branched off at a more basal split within crustaceans [88,93], forming a sister group relationship to Ostracoda and contrary they branched off at a more terminal split in the non-stationary tree we cannot draw a final conclusion about the placement of Malacostraca. Unfortunately the position of the Pentastomida remains ambiguous in our analyses, we argue that low pP values might be induced by conflicting phylogenetic signal.

#### Sister group of Hexapoda

The sister group of Hexapoda is still disputed. Most molecular studies support paraphyly of crustaceans with respect to hexapods. A sister group relationship between Branchiopoda and Hexapoda was proposed for the first time by Regier and Shultz [94], yet with low support. Shultz and Regier [5] and Regier et al. [12] corroborated this relationship, which is likewise favored by authors of rRNA-based studies [1,11], despite their result that Cyclopidae (Copepoda) is the sister group of Hexapoda. Our denser taxon sampling further supports Copepoda as the sister group to Hexapoda, but the low support value might indicate conflicting signal. This clade up till now, however, lacks any support from morphological studies.

#### Ancient splits within pterygote insects

We find that the rRNA data cannot robustly resolve the most ancient splits within Pterygota. Nonetheless, rRNA data, when analyzed under more realistic models favour Chiastomyaria as the most likely hypothesis. Since all three possible arrangements of Odonata, Ephemeroptera and Neoptera likewise receive morphological support, we agree with Whitfield and Kjer [35] that the ambiguity can best be explained by early 'explosive radiation' within Pterygota.

## Conclusion

We conclude that the implementation of biologically realistic model parameters, such as site interaction (mixed DNA/RNA models) and compositional heterogeneity of base frequency, is fundamental to robustly reconstruct phylogenies. The most conspicuous examples comparing our tress are a) the position of *Hutchinsoniella *(Crustacea), although a low pP value of 0.59 in the non-stationary tree prohibits conclusions about its internal crustacean relationship and b) the well supported position of *Ctenolepisma *and *Lepisma *(Zygentoma). As a consequence, the monophyly of Hexapoda, Entognatha and Ectognatha and Dicondylia received support only in the time-heterogeneous approach. Several artificial clades remain in our analyses which cannot be causally explained unambiguously. However, the examples given here clearly demonstrate that the probability to causally explain some incongruities between different data sets, as well as the correction of certain obvious misplacements, is enhanced by using more complex but realistic models. The present study aimed to incorporate background knowledge on the evolution of molecular sequences in general and ribosomal RNA-genes in special into various steps of data processing. For all steps fully automated methods were used, including an automated secondary structure guided alignment approach, a software that enables to distinguish random similarity from putative phylogenetic signal, mixed models that avoid artefacts due to co-variation among sites, and analyses that account for variation of evolutionary rates among lineages. The resolution of many relationships among arthropods, and the minimization of obvious misplacements demonstrate that the increased computational effort pays off.

## Methods

### Taxon Sampling

Our taxon sampling was designed to represent a taxonomically even collection of specimens across arthropod groups. In particular, we took care to include taxa which do not differ too widely from the hypothetical morphological ground-pattern of the represented group, when possible [53,78]. In total we included 148 concatenated 18S and 28S rRNA sequences in the analysis (Additional file 1). Of these, we contributed 103 new sequences, 41 for the 18S and 62 for the 28S rRNA gene, respectively. Only sequences which span at least 1500 bp for the 18S and 3000 bp for the 28S were included. For 29 taxa we had to construct chimeran concatenated sequences of 18S and 28S rRNA sequences of different species, marked with an asterisk. Details are listed in Additional file 5, we chose species as closely related as possible depending on it's availability in GenBank. The outgroup included the concatenated 18S and 28S rRNA sequences of *Milnesium *sp. (Tardigrada).

### Laboratory work

Collected material was preserved in 94 – 99% ethanol or liquid nitrogen. Samples were stored at temperatures ranging from -20°C to -80°C. DNA extraction of complete specimens or tissue followed different standard protocols. We used phenol-chloroform isoamyl extraction [95], standard column DNA extraction kits DNeasy Blood & Tissue Kit (Qiagen) and NucleoSpin Tissue Kit (Machery-Nagel) following the manual. Single specimens were macerated for extraction, only specimens of *Ctenocephalides felis *were pooled. Manufacturer protocols were modified for all crustaceans, some apterygote hexapods and myriapods (overnight incubation and adding 8 *μ*l RNAse [10 mg/ml] after lysis). Extracted genomic DNA was amplified with the Illustra GenomiPhi V2 DNA Amplification Kit (GE Healthcare) for tiny, rare or hard to collect specimens.

Partly published rRNA primer sets were used, they were designed in part for specific groups (Additional file 6 and 7). The 18S of crustaceans was amplified in one PCR product and sequenced using four primer combinations. The 18S of apterygotes was amplified in three or four fragments (Additional file 8). The 28S of crustaceans and basal hexapods was amplified in nine overlapping fragments starting approximately in the middle of the rRNA 5.8S to the nearly end of the D12 of 28S rRNA (Additional file 9). The 28S of odonats was amplified in seven or eight, the 28S of ephemeropterans and neopterans in eight overlapping fragments (Additional file 10). Primers were ordered from Metabion, Biomers or Sigma-Genosys. PCR products were purified using following kits: NucleoSpin ExtractionII (Machery-Nagel), QIAquick PCR purification kit (Qiagen), peqGOLD Gel Extraction Kit (peqLab Biotechnologie GmbH), MultiScreen PCR Plate (Millipore) and ExoI (Biolabs Inc.)/SAP (Promega). Some samples were purified using a NHAc [4 mol] based ethanol precipitation. In case of multiple bands fragments with the expected size were cut from 1% – 1.5% agarose gel and purified according to manufacturer protocols.

Cycle sequencing and sequence analyses took place on different thermocyclers and sequencers. Cycle sequencing products were purified and sequenced double stranded. Several amplified and purified PCR products were sequenced by Macrogen (Inc.), Korea. Sequencing of the 28S fragment 28V – D10b.PAUR of the Pauropodidae sp. (Myriapoda) was only successful via cloning. Fragments of the 28S rRNA of the diplopod *Monographis *sp. (Myriapoda) were processed following Mallatt et al. [11] and Luan et al. [44]. Please refer to the electronic supplement (Additional file 11) for detailed information about PCR-conditions, applied temperature profiles (Additional file 12), primer combinations, used chemicals (Additional file 13) and settings to amplify DNA fragments. Sequence electropherograms were analyzed and assembled to consensus sequences applying the software SeqMan (DNAStar Lasergene) or BioEdit 7.0 [96]. All sequences or composed fragments were blasted in NCBI using BLASTN, mega BLAST or "BLAST 2 SEQUENCES" [97] to exclude contaminations.

### Alignments and alignment evaluation

Secondary structures of rRNA genes were considered (as advocated in [98-101] to improve sequence alignment. Structural features are the targets of natural selection, thus the primary sequence may vary, as long as the functional domains are structurally retained. Alignments and their preparation for analyses were executed for each gene separately. We prealigned sequences using MUSCLE v3.6 [102]. Sequences of 24 taxa of Pterygota were additionally added applying a profile-profile alignment [103]. The 28S sequences of *Hutchinsoniella macracantha *(Cephalocarida), *Speleonectes tulumensis *(Remipedia), *Raillietiella *sp. (Pentastomida), *Eosentomon *sp. (Protura) and *Lepisma saccharina *(Zygentoma) were incomplete. Apart from *L. saccharina*, prealignments of these taxa had to be corrected manually. We used the "BLAST 2 SEQUENCES" tool to identify the correct position of sequence fragments in the multiple sequence alignment (MSA) for these incomplete sequences.

The software RNAsalsa [56] is a new approach to align structural RNA sequences based on existing knowledge about structure patterns, adapted constraint directed thermodynamic folding algorithms and comparative evidence methods. It automatically and simultaneously generates both individual secondary structure predictions within a set of homologous RNA genes and a consensus structure for the data set. Successively sequence and structure information is taken into account as part of the alignment's scoring function. Thus, functional properties of the investigated molecule are incorporated and corroborate homology hypotheses for individual sequence positions. The program employs a progressive multiple alignment method which includes dynamic programming and affine gap penalties, a description of the exact algorithm of RNAsalsa will be presented elsewhere.

As a constraint, we used the 28S + 5.8S (U53879) and 18S (V01335) sequences and the corresponding secondary structures of *Saccharomyces cerevisae *extracted from the European Ribosomal Database [104-106]. Structure strings were converted into dot-bracket-format using Perl-scripts. Folding interactions between 28S and 5.8S [74,107,108] required the inclusion of the 5.8S in the constraint to avoid artificial stems. Alignment sections presumably involved in the formation of pseudoknots were locked from folding to avoid artifacts. Pseudoknots in *Saccharomyces cerevisae *are known for the 18S (stem 1 and stem 20, V4-region: stem E23 9, E23 10, E23 11 and E23 13) while they are lacking in the 28S secondary structure. Prealignments and constraints served as input, and RNAsalsa was run with default parameters. We constructed manually chimeran 18S sequences of *Speleonectes tulumensis *(EU370431, present study and L81936) and 28S sequences of *Raillietiella *sp. (EU370448, present study and AY744894). Concerning the 18S of *Speleonectes tulumensis *we combined positions 1–1644 of L81936 and positions 1645–3436 of sequence EU370043. Regarding the 28S of *Raillietiella *we combined positions 1–3331 of AY744894 with positions 3332–7838 of sequence EU370448. Position numbers refer to aligned positions.

RNAsalsa alignments were checked with ALISCORE[65]. ALISCORE generates profiles of randomness using a sliding window approach. Sequences within this window are assumed to be unrelated if the observed score does not exceed 95% of scores of random sequences of similar window size and character composition generated by a Monte Carlo resampling process. ALISCORE generates a list of all putative randomly similar sections. No distinction is made between random similarity caused by mutational saturation and alignment ambiguity. A sliding window size (*w *= 6) was used, and gaps were treated as ambiguities (- N option).

The maximum number of possible random pairwise comparisons (- r: 10,878) was analyzed. After the exclusion of putative random sections and uninformative positions using *PAUP *4.0b10, alignments were checked for compositional base heterogeneity using the *χ*^2^-test. Additionally, for each sequence the heterogeneity-test was performed for paired and unpaired sites separately. Further heterogeneity-tests were applied to determine the minimal number of base frequency groups.

RNAsalsa generated consensus structure strings for 18S and 28S rRNA sequences, subsequently implemented in the MSA. Randomly similar sections identified by ALISCORE were excluded using a Perl-script. ALISCORE currently ignores base pairings. If ambiguously aligned positions within stems are discarded the corresponding positions will be handled as an unpaired character in the tree reconstruction. The cleaned 18S and 28S alignments were concatenated.

To analyze information content of raw data SplitsTree4 was used to calculate phylogenetic networks (see Huson and Bryant [109] for a review of applications). We compared the network structure based on the neighbornet algorithm [110] and applying the LogDet transformation, e.g. [111,112]. LogDet is a distance transformation that corrects for biases in base composition. The network graph gives a first indication of signal-like patterns and conflict present in the alignments. We used the alignment after filtering of random-like patterns with ALISCORE.

### Phylogenetic reconstruction

Mixed DNA/RNA substitution models were chosen, in which sequence partitions corresponding to loop regions were governed by DNA models and partitions corresponding to stem regions by RNA models that consider co-variation. Among site rate variation [113] was implemented in both types of substitution models. Base frequency tests indicated that base composition was inhomogeneous among taxa (see results), suggesting non-stationary processes of sequence evolution. To take such processes into account the analyses were performed in *PHASE-2.0 *[63] to accommodate this compositional heterogeneity to minimize bias in tree reconstruction. Base compositional heterogeneity is implemented in *PHASE-2.0 *according to the ideas developed by Foster [87].

We limited the number of candidate models to the REV + Γ, TN93 + Γ and the HKY85 + Γ models for loop regions and the corresponding RNA16I + Γ, RNA16J + Γ and RNA16K + Γ models for stem regions. Site heterogeneity was modeled by a discrete gamma distribution [114] with six categories. The extent of invariant characters was not estimated since it was shown to correlate strongly with the estimation of the shape parameter of the gamma distribution [113,115-117]. The data was partitioned into four units representing loop and stem regions of 18S rRNA and loop and stem regions of 28S rRNA. DNA and RNA substitution model parameters were independently estimated for each partition. Substitution models were selected based on results of time-homogeneous setups. We tested three different combinations of substitution models, REV + Γ & RNA16I + Γ, TN93 + Γ & RNA16J + Γ and HKY85 + Γ & RNA16K + Γ. We used Dirichlet distribution for priors, proposal distribution and Dirichlet priors and proposals for a set of exchangeability parameters (Additional file 14) described in Gowri-Shankar and Rattray [60].

Appropriate visiting of the parameter space according to the posterior density function [118] was checked by plotting values of each parameter and monitoring their convergence. This was calculated for all combinations after 500,000 generations (sampling period: 150 generations). We discarded models in which values of several parameters did not converge. For models which displayed convergence of nearly all parameter values, we re-run MCMC processes with 3,000,000 generations and a sampling period of 150 generations. Prior to comparison of the harmonic means of *lnL *values, 299,999 generations were discarded as burn-in. After a second check for convergence the model with the best fitness was selected applying a Bayes Factor Test (BFT) to the positive values of the harmonic means calculated from *lnL *values [67,68]. The favored model (2*lnB*_10 _> 10) was used for final phylogenetic reconstructions.

To compare results of time-homogeneous and time-heterogeneous models, 14 independent chains of 7,000,000 generations and two chains of 10 million generations for both setups were run on Linux clusters (Pentium 4, 3.0 GHz, 2 Gb RAM, and AMD Opteron Dual Core, 64 bit systems, 32 Gb RAM). For each chain the first two million generations were discarded as burn-in (sampling period of 1000). The setup for the time-homogeneous approach was identical to the pre-run except for number of generations, sampling period and burn-in. The setting for the time-heterogeneous approach differed (Additional file 4). We followed the method of Foster [87] and Gowri-Shankar and Rattray [60] in the non-homogeneous setup whereby only a limited number of composition vectors can be shared by different branches in the tree. Exchangeability parameters (average substitution rate ratio values, rate ratios and alpha shape parameter) were fixed as input values. Values for these parameters were computed from results of the preliminary time-homogeneous pre-run (3,000,000 generations). A consensus tree was inferred in *PHASE mcmcsummarize *using the output of the pre-run. This consensus tree topology and the model file of this run served as input for a ML estimation of parameters in *PHASE optimizer*. Estimated values of exchangeability parameters from the resulting *optimizer *output file and estimated start values for base frequencies were fed into *mcmcphase *for the time-heterogeneous analysis. Values of exchangeability parameters remained fixed during the analysis. The number of allowed base frequency categories (models) along the tree was also fixed. The number of base frequency groups was set to three "submodels"), reflecting base frequency heterogeneity.

Harmonic means of *lnL *values of these 16 independent chains were again compared with a BFT to identify possible local optima in which a single chain might have been trapped. We only merged sample data of chains with a 2*lnB*_10_-value < 10 [67] using a Perl-script to construct a "metachain" [119]. Finally we included ten time-heterogeneous chains and three time-homogeneous chains. The assembled meta-chains included 56 million generations for the non-stationary approach (Additional file 15) and 18 million generations for the time-homogeneous approach (Additional file 16), burn-ins were discarded. Consensus trees and posterior probability values were inferred using *mcmcsummarize*. Branch lengths of the time-homogeneous and time-heterogeneous consensus tree were estimated using three *mcmcphase *chains (4 million generations, sampling period 500, topology changes turned off, starting tree = consensus tree, burn-in: 1 million generations) from different initial states with a Gowri-Shankar modified *PHASE *version. To infer mean branch lengths we combined data with the described branch lengths and *mcmcsummarize*. These mean branch lengths were used to redraw the consensus tree (Additional file 4).

Localities of sampled specimen used for amplication are listed in Additional file 17.

## List of abbreviations

rRNA: ribosomal RNA; PCR: polymerase chain reaction; RNA: ribonucleic acid; DNA: deoxyribonucleic acid; df: degree of freedom; P: probability; pP: posterior probability; sp.: species epithet not known; *ln*: natural logarithm or *log*_*e*_; BFT: Bayes Factor Test.

## Authors' contributions

BMvR, KM and BM conceived the study, designed the setup and performed all analyses. VG complemented *PHASE-2.0 *and contributed to *PHASE-2.0 *analyses setup. RRS, HOL, BM provided RNAsalsa and software support. JWW allocated the neighbornet-analysis. BMvR, KM, ED, SS, HOL, DB and YL contributed sequence data and designed primers. BMvR, KM, BM, NUS and JWW wrote the paper with comments and revisions from ED, VG, RRS, DB, SS, GP, HH and YL. All authors read and approved the final manuscript.

## Supplementary Material

Additional file 1 **Taxa list**. Taxa list of sampled sequences. * indicates concatenated 18S and 28S rRNA sequences from different species. For combinations of genes to construct concatenated sequences of chimeran taxa, see Table S1. ** contributed sequences in the present study (author of sequences). Click here for file

Additional file 2 **LogDet corrected network of concatenated 18S and 28S rRNA alignment**. LogDet corrected network plus invariant site models (30.79% invariant sites) using SplitsTree4 based on the concatenated 18S and 28S rRNA alignment after exclusion of randomly similar sections evaluated with ALISCORE. Click here for file

Additional file 3 **Bayesian support values for selected clades**. List of Baysian support values (posterior probability, pP) for selected clades of the time-heterogeneous and time-homogeneous tree. Click here for file

Additional file 4 **Detailed flow of the analysis procedure in the software package PHASE-2.0**. Options used in *PHASE-2.0 *are italicized above the arrows and are followed by input files. Black arrows represent general flows of the analysis procedure, green arrows show that results or parameter values after single steps were inserted or accessed in a further process. Red block-arrows mark the final run of the time-heterogeneous and time-homogeneous approach with 16 chains each (2 × 118,000,000 generations). **First row**: I.) We prepared 3 control files (control.mcmc) for *mcmcphase *using three different mixed models. This "pre-run" was used for a first model selection (500,000 generations for each setting). We excluded model (C) based on non-convergence of parameter values. II.) We repeated step one (I.) with 3,000,000 generations using similar control files (different number of generations and random seeds) of the two remaining model settings. Calculated ln likelihoods values of both chains were compared in a BFT resulting in the exclusion of mixed model (A). Parameter values of the remaining model (B) were implemented in the time-heterogeneous setting. III.) We started the final analysis (final run) using sixteen chains for both the time-homogeneous and the time-heterogeneous approach. In the final time-homogeneous approach, the control files were similar to step II.) except for a different number of generations and random seeds. **Second row**: Additional steps were necessary prior to the computation of the final time-heterogeneous chains. We applied *mcmcsummarize *for the selected mixed model (B) to calculate a consensus tree. *Optimizer *was executed to conduct a ML estimation for each parameter value (opt.mod) based on the inferred consensus tree and optimized parameter-values (mcmc-best.mod), a data file delivered by *mcmcphase*. Estimated values were implemented in an initial.mod file. The initial.mod file and its parameter values were accessed by the control files of the final time-heterogeneous chains (only topology and base frequencies estimated). **Third row**: Trees were reconstructed separately for the time-homogeneous and time-heterogeneous setting. All chains of each approach were tested in a BFT against the chain with the best *lnL*. We only included chains with a 2*lnB*_10_-value > 10. From these chains we constructed a metachain for each setting using Perl and applied *mcmcsummarize *to infer the consensus topology. To estimate branch lengths properly we ran *mcmcphase*, resulting branch lengths were implemented in the consensus trees. Finally, both trees were optimized using graphic programs (Dendroscope, Adobe Illustrator CS II). Click here for file

Additional file 5 **List of chimeran species for concatenated 18S and 28S rRNA sequences** Click here for file

Additional file 6 **Primer list 18S rRNA** Click here for file

Additional file 7 **Primer list 28S rRNA** Click here for file

Additional file 8 **Primercard of the 18S rRNA gene for hexapods, myriapods and crustaceans**. Primers used for hexapods or myriapods are shown in the upper part, primers for crustaceans in the lower part. Positions of forward primers are marked with green arrows, those of reverse primers with red arrows. When different primers with identical position were used, all primer labels are given at the single arrow for the specific position. Primers and their combinations are given in Additional file 6 and 11. Click here for file

Additional file 9 **Primercard of the 28S rRNA gene for crustaceans, hexapods and myriapods**. Positions of forward primers are tagged with green arrows, those of reverse primers with red arrows. When different primers with identical position were used, all primer labels are given at the single arrow for the specific position. Primers and their combinations are given in Additional file 7 and 11. Click here for file

Additional file 10 **Primercard of the 28S rRNA gene for pterygots**. Positions of forward primers are assigned by green arrows, those of reverse primers with red arrows. When different primers with identical position were used, all primer labels are given at the single arrow for the specific position. Primers and their combinations are given in Additional file 7 and 11. Click here for file

Additional file 11 **Supplementary Information**. Supplementary information for lab work (amplificaion, purification and sequencing of PCR products). Click here for file

Additional file 12 **PCR temperature-profiles** Click here for file

Additional file 13 **PCR chemicals** Click here for file

Additional file 14 **Setting of exchangeability parameters used in pre-runs**. Listed settings of exchangeability parameters used in pre-runs in *PHASE-2.0*. Click here for file

Additional file 15 **Included chains to infer the time-heterogeneous consensus tree**. Number of chains, generations per chain, harmonic means (*lnL*) and 2*lnB*_10_-values included to infer the time-heterogeneous consensus tree. Click here for file

Additional file 16 **Included chains to infer the time-homogeneous consensus tree**. Number of chains, generations per chain, harmonic means (*lnL*) and 2*lnB*_10_-values included to infer the time-homogeneous consensus tree. Click here for file

Additional file 17 **Localities of sampled taxa** Click here for file
