# Supplementary figures and images for: Can comprehensive background knowledge be incorporated into substitution models to improve phylogenetic analyses? A case study on major arthropod relationships
Source: BMC Evol Biol. 2009 May 27;9:119. doi: 10.1186/1471-2148-9-119 (PMC2695459; doi:10.1186/1471-2148-9-119)

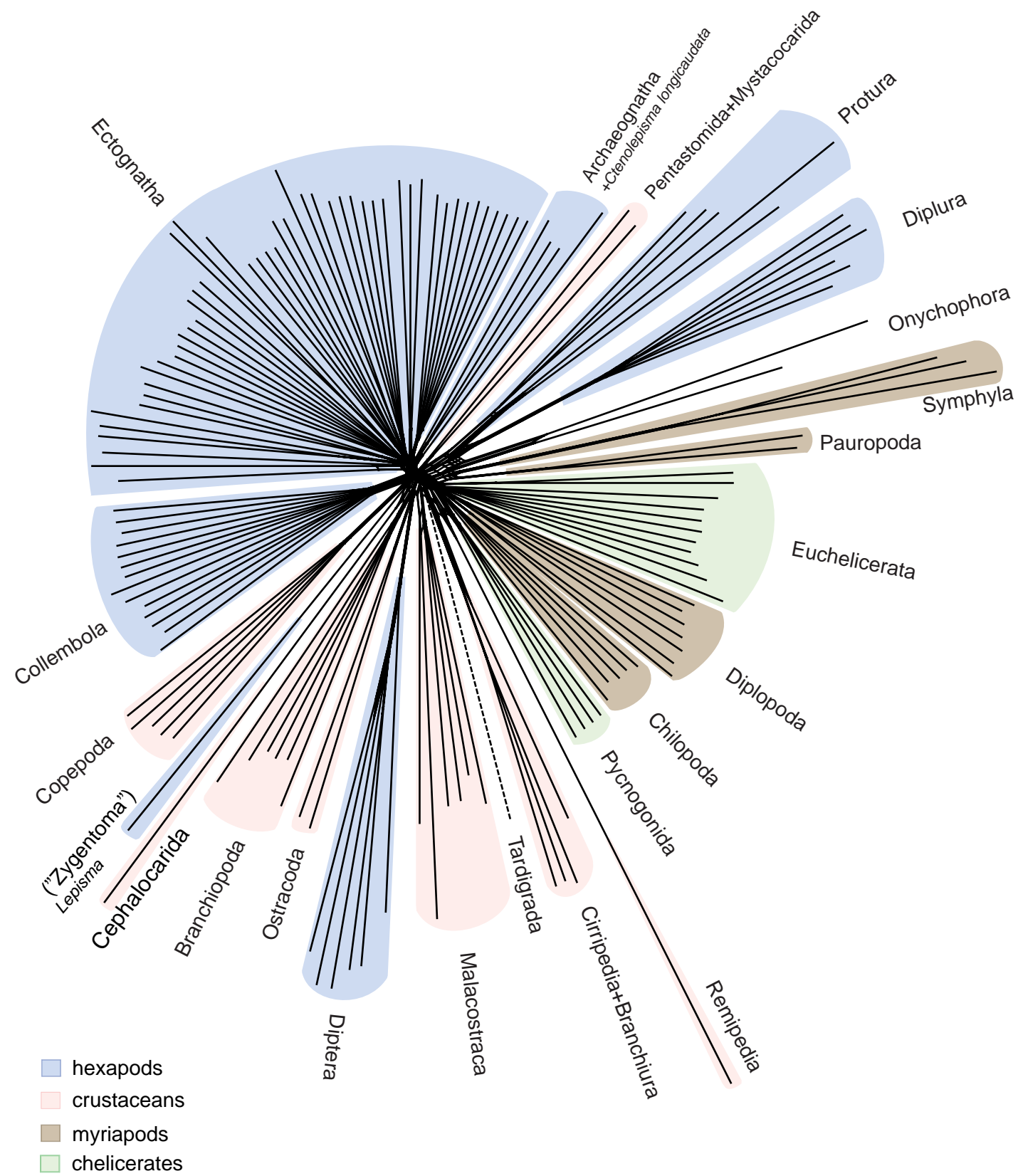

Supplement: Additional file 2 — LogDet corrected network of concatenated 18S and 28S rRNA alignment. LogDet corrected network plus invariant site models (30.79% invariant sites) using SplitsTree4 based on the concatenated 18S and 28S rRNA alignment after exclusion of randomly similar sections evaluated with ALISCORE. [file 1471-2148-9-119-S2.pdf]

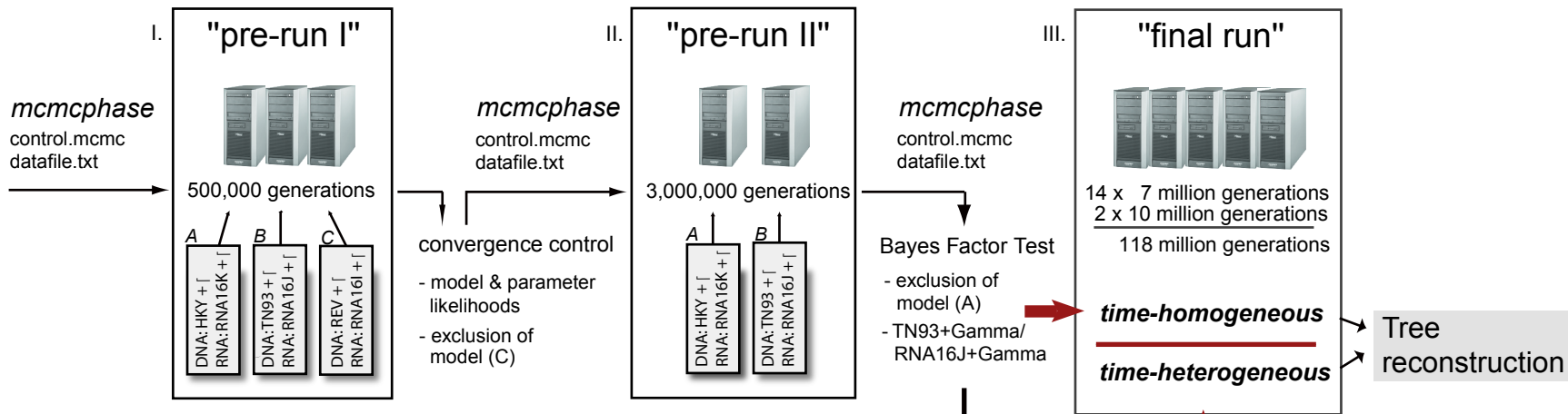

## Procedure time-heterogeneous setting

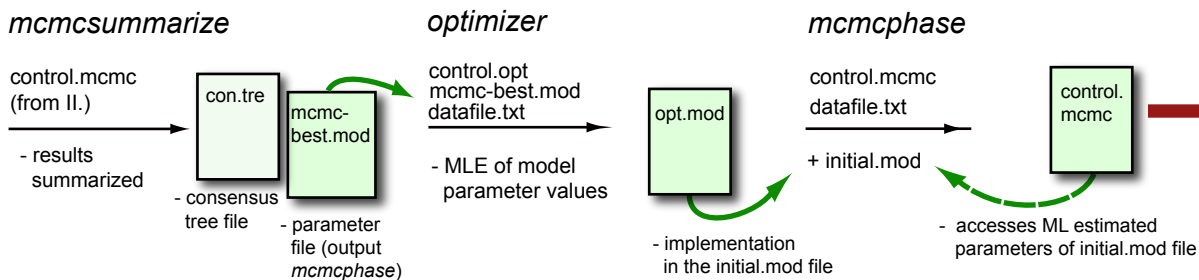

## Tree reconstruction

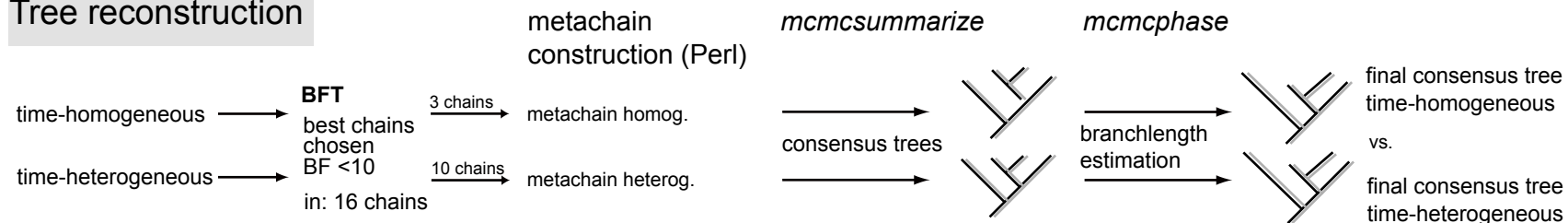

Supplement: Additional file 4 — Detailed flow of the analysis procedure in the software package PHASE-2.0. Options used in PHASE-2.0 are italicized above the arrows and are followed by input files. Black arrows represent general flows of the analysis procedure, green arrows show that results or parameter values after single steps were inserted or accessed in a further process. Red block-arrows mark the final run of the time-heterogeneous and time-homogeneous approach with 16 chains each (2 × 118,000,000 generations). First row: I.) We prepared 3 control files (control.mcmc) for mcmcphase using three different mixed models. This "pre-run" was used for a first model selection (500,000 generations for each setting). We excluded model (C) based on non-convergence of parameter values. II.) We repeated step one (I.) with 3,000,000 generations using similar control files (different number of generations and random seeds) of the two remaining model settings. Calculated ln likelihoods values of both chains were compared in a BFT resulting in the exclusion of mixed model (A). Parameter values of the remaining model (B) were implemented in the time-heterogeneous setting. III.) We started the final analysis (final run) using sixteen chains for both the time-homogeneous and the time-heterogeneous approach. In the final time-homogeneous approach, the control files were similar to step II.) except for a different number of generations and random seeds. Second row: Additional steps were necessary prior to the computation of the final time-heterogeneous chains. We applied mcmcsummarize for the selected mixed model (B) to calculate a consensus tree. Optimizer was executed to conduct a ML estimation for each parameter value (opt.mod) based on the inferred consensus tree and optimized parameter-values (mcmc-best.mod), a data file delivered by mcmcphase. Estimated values were implemented in an initial.mod file. The initial.mod file and its parameter values were accessed by the control files of the final time-heterogen [file 1471-2148-9-119-S4.pdf]

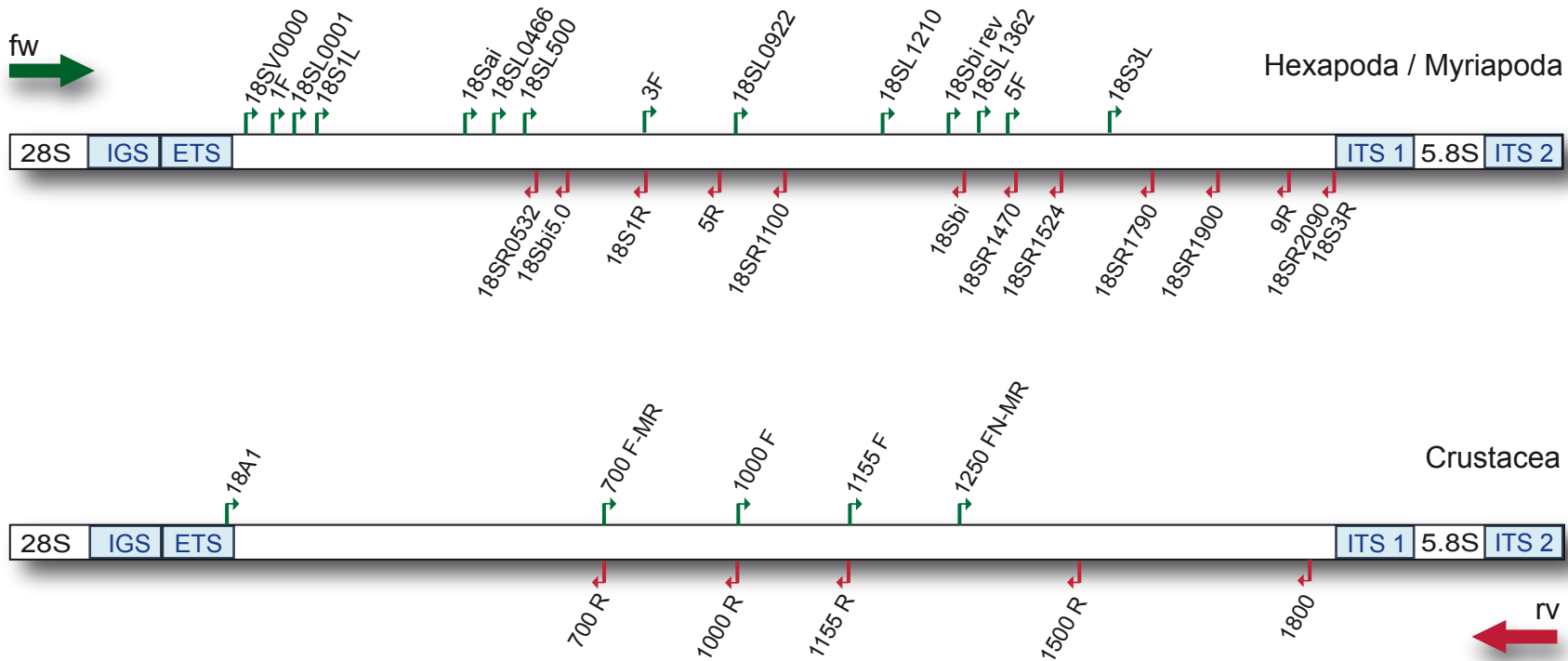

Supplement: Additional file 8 — Primercard of the 18S rRNA gene for hexapods, myriapods and crustaceans. Primers used for hexapods or myriapods are shown in the upper part, primers for crustaceans in the lower part. Positions of forward primers are marked with green arrows, those of reverse primers with red arrows. When different primers with identical position were used, all primer labels are given at the single arrow for the specific position. Primers and their combinations are given in Additional file 6 and 11. [file 1471-2148-9-119-S8.pdf]

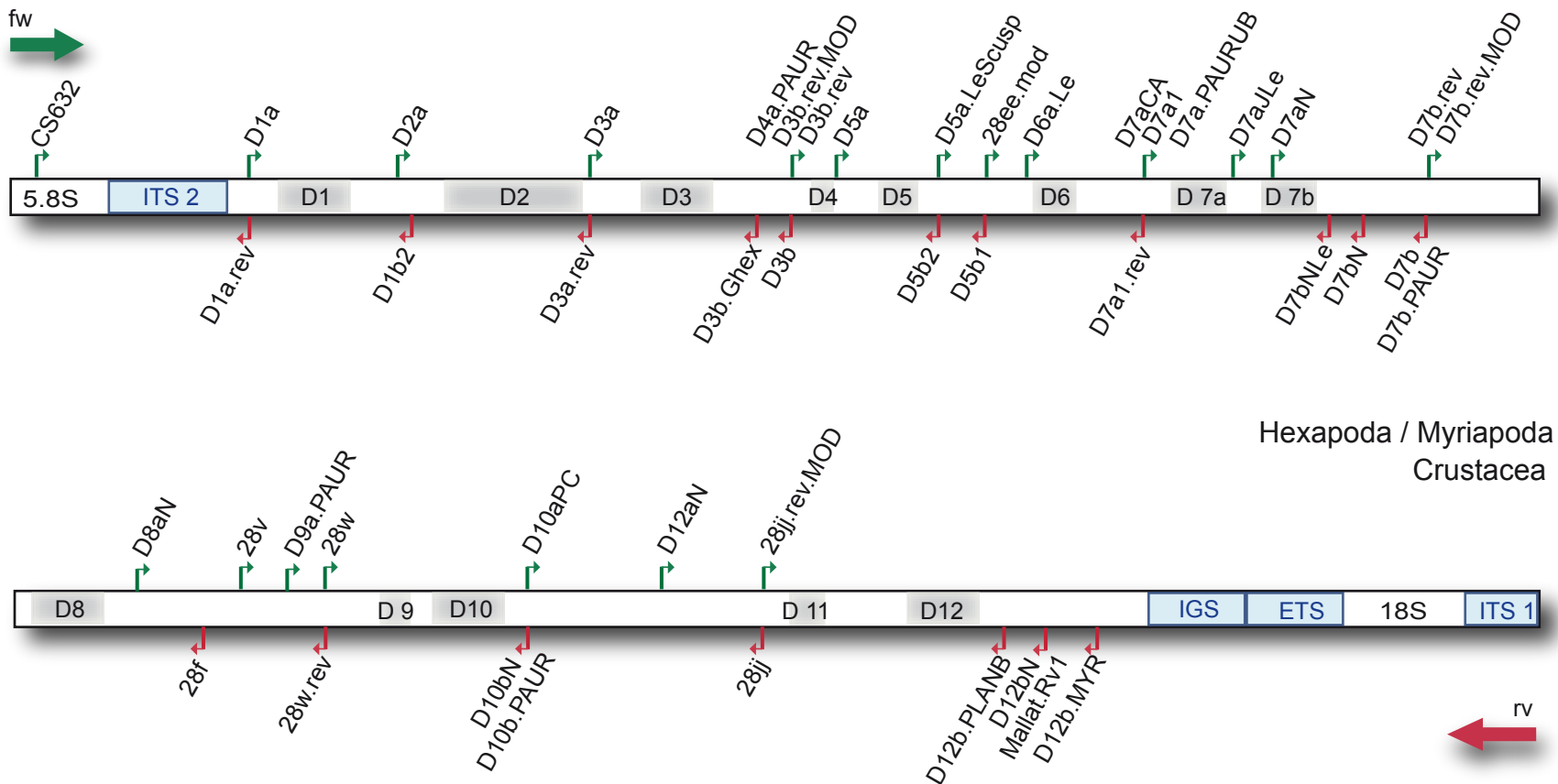

Supplement: Additional file 9 — Primercard of the 28S rRNA gene for crustaceans, hexapods and myriapods. Positions of forward primers are tagged with green arrows, those of reverse primers with red arrows. When different primers with identical position were used, all primer labels are given at the single arrow for the specific position. Primers and their combinations are given in Additional file 7 and 11. [file 1471-2148-9-119-S9.pdf]

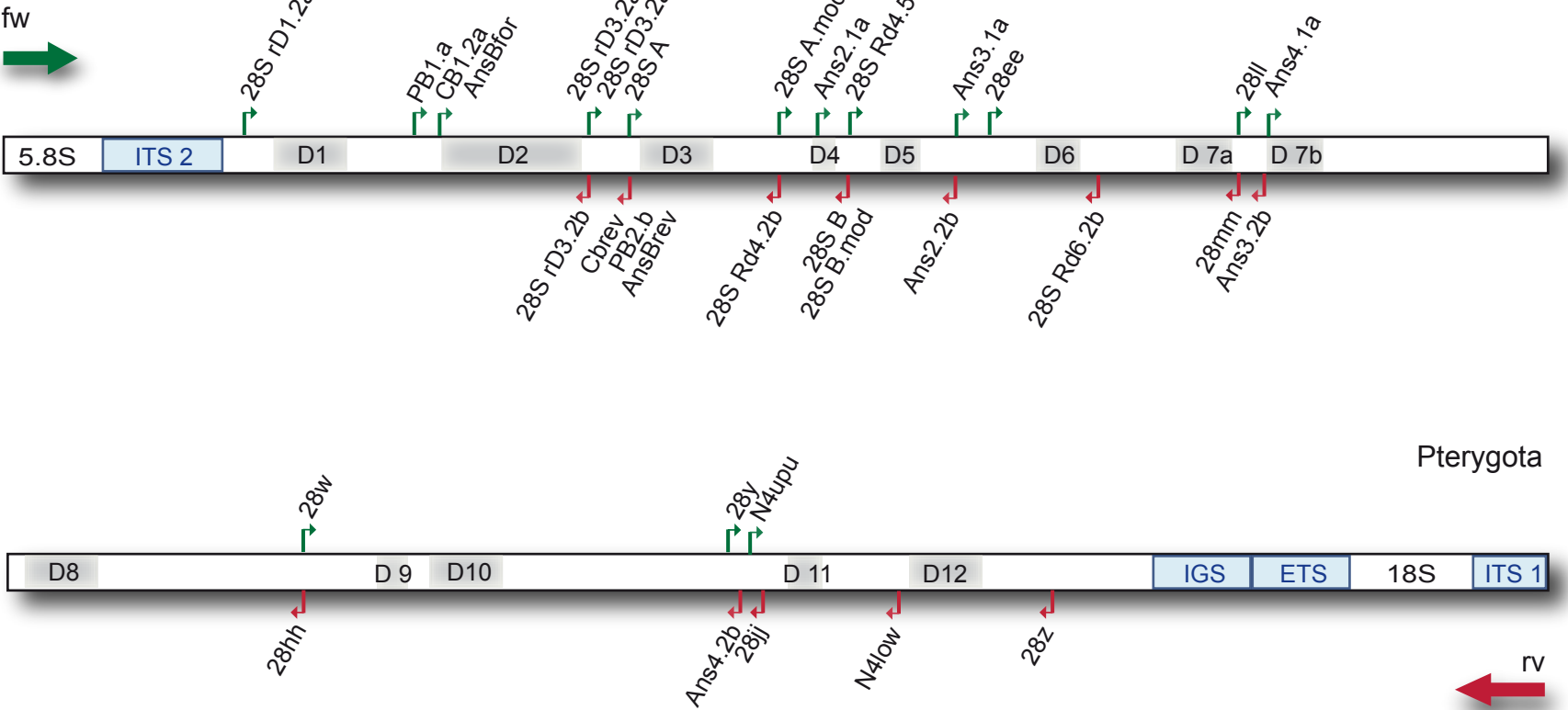

Supplement: Additional file 10 — Primercard of the 28S rRNA gene for pterygots. Positions of forward primers are assigned by green arrows, those of reverse primers with red arrows. When different primers with identical position were used, all primer labels are given at the single arrow for the specific position. Primers and their combinations are given in Additional file 7 and 11. [file 1471-2148-9-119-S10.pdf]
