# Supplementary material for: Can comprehensive background knowledge be incorporated into substitution models to improve phylogenetic analyses? A case study on major arthropod relationships
Source: BMC Evol Biol. 2009 May 27;9:119. doi: 10.1186/1471-2148-9-119 (PMC2695459; doi:10.1186/1471-2148-9-119)
Supplement: Additional file 11 — Supplementary Information. Supplementary information for lab work (amplificaion, purification and sequencing of PCR products). [file 1471-2148-9-119-S11.pdf]

Can comprehensive background knowledge be incorporated into substitution models to improve phylogenetic analyses? A case study on major arthropod relationships: Supplementary Information

Björn M von Reumont*[1], Karen Meusemann[1] , Nikolaus U Szucsich[2] , Emiliano Dell'Ampio[2] , Vivek Gowri-Shankar , Daniela Bartel[2] , Sabrina Simon[3] , Harald O Letsch[1] , Roman R Stocsits[1] , Yun-xia Luan[4] , Johann Wolfgang Wägele[1] , Günther Pass[2] , Heike Hadrys[3,5], and Bernhard Misof[1,6]

[1]Molecular Lab, Zoologisches Forschungsmuseum A. Koenig, Bonn, Germany
[2]Department of Evolutionary Biology, University Vienna, Vienna, Austria
[3]ITZ, Ecology & Evolution, Stiftung Tieraerztliche Hochschule Hannover, Hannover, Germany
[4]Institute of Plant Physiology and Ecology, Shanghai Institutes for Biological Sciences, Chinese Academy of Sciences, Shanghai, China
[5]Department of Ecology and Evolutionary Biology, Yale University, New Haven, CT, USA
[6]UHH Biozentrum Grindel und Zoologisches Museum, University of Hamburg, Hamburg, Germany

Email: Bjoern von Reumont*- bmvr@arcor.de; Karen Meusemann - mail@karen-meusemann.de; Nikolaus U Szucsich - nikola.szucsich@univie.ac.at; Emiliano Dell'Ampio - emiliano.dell.ampio@univie.ac.at; Vivek Gowri-Shankar - gowrishv@cs.man.ac.uk; Daniela Bartel - dani.bartel@chello.at; Sabrina Simon - sabrina.simon@ecolevol.de; Harald O Letsch - hletsch@freenet.de; Roman R Stocsits - roman.stocsits@gmail.com; Yun-xia Luan - yxluan@sibs.ac.cn; Johann Wolfgang Wägele - w.waegele.zfmk@uni-bonn.de; Günther Pass - guenther.pass@univie.ac.at; Heike Hadrys - heike.hadrys@ecolevol.de; Bernhard Misof - bernhard.misof@uni-hamburg.de;

*Corresponding author

Amplification of PCR products

Unless otherwise noted all applied protocols refer to manufacturers advices. All used primers are given in Additional file 9 and 10 [1–18]. Splitting the 18S rRNA of apterygote hexapods and myriapods in three or four fragments following primer settings were used: A) 1F/5R, 3F/18Sbi, 5F/9R B) 18S L 0001/18S R 0532, 18S L 0466/18S R 1100, 18S L 0922/18S R 1524, 18S L 1362/18S R 2090 C) 18S 1 L/18S 1 R, 18S L 500/18S R 1470, 18S L 1210/ 18S R 1790, 18S 3 L/18S 3 R D) 18SV0000/18Sbi5.0, 18Sai/18Sbi or alternative 18Sai/18SR1900, 18Sbi-rev/18SR1900. We combined primer pairs of primer sets A, B, C and D to amplify the complete 18S. The 18S in crustaceans was amplified in one PCR product (18A1/1800) and sequenced with eight primers (700 F, 1000 F, 1155 F, 1250 FN, 700 R, 1000 R, 1155 R and 1500 R), see Additional file 8. The PCR-Multiplex-Kit (Qiagen) was used to prevent pooling of weak PCR products. HotstartPCRs [19] for 18S apterygote hexapods and myriapods were more successfully (Additional file 12). The nuclear 28S rRNA gene of crustaceans, apterygote hexapods and myriapods was amplified in nine overlapping fragments using following primer combinations: CS632/D1b2, D1a/D3b, D2a/D3a.rev, D3a/D5b1, D5a/D7b, D7a1/28f, D7b.rev/28w.rev, 28v–28jj and D12aN/ D12bN or alternatively D12aN/ D12bPLANB, D12aN/D12bMYR or D12aN/MallatRv1. Different primer combinations were used whenever necessary for specific taxa. This was essential for the divergent domain D12. Alternative combinations for crustaceans are: D3b.rev/D5b2,

D3b.rev/D5b1, D1a/D5b1, D1a/D5b2, D2a/D5b1, D2a/D5b2, D3a/D5b2, D7aN/28f, D7b.rev/28f, D7brev/D10bN, D10aPC/D12bN and D12aN/D12b.PLANB. To complete the 28S for *Eosentomon sakura* following primer combinations were necessary: D1a/D1b2, D2a/D3a.rev, D2a/D3b and 28ee.mod/D7b (Additional file 9). Based on two sets of universal primers [10, 12], specific primers were designed to amplify 28S sequences of Odonata in seven overlapping fragments. The conserved part of the 5'–end was covered by the universal primer sets Rd1.2a/rD3.2a and 28 A/28 B (Additional file 10), interrupted by the highly variable regions D2 and D3, for which three specifically adapted primer sets were used: AnsBfor/AnsBrev for standard, CB1.2a/CBrev for Libellulidae and PB1.a/PB2.b for some taxa in Aeshnoidea. The domains II – IV were covered by three specific primer sets: Ans2.1a/Ans2.2b, Ans3.1a/Ans3.2b and Ans4.1a/Ans4.2b, respectively. At the 3'-end the primers N4Lfor/N4Lrev amplified the conserved region before the variable D12 domain (Additional file 7). 28S PCR-products for Pterygota were amplified with following primer combinations: 28S rD1.2a/28S Rd4.2b, 28S rD3.2a/28S B, 28S A/28S Rd6.2b, 28y/28z, 28ll/28hh and 28w/28jj (Additional file 10). The amount of Taq polymerase (Bioline) was increased to 0.3 μl, respectively the amount of sterile H_2O was diminished for the two last mentioned primer settings (Additional file 13). For the primer pairs 28ee/28hh and 28ll/28jj reagents (Roche) were used with a different composition of PCR-mix (Additional file 13).

Purification and Sequencing reactions

Unless otherwise noted all applied protocols refer to manufacturers advices; weak PCR products were pooled for purificaton. 18S rRNA of apterygotes and myriapods, 28S rRNA of crustaceans and odonates: Products were purified with the NucleoSpin Extract II (Macherey-Nagel) or with enzymes ExoI/SAP. 0.12 μl ExoI (20 u/μl,Biolabs), 0.45 μl SAP (Shrimp Alkaline Phosphatase, 1 u/μ, Promega) band 2.43 μl RNAse-free sterile water was mixed on ice. 3 μl of the mixture was added to 10 μl of PCR product and incubated for 15 min. at 37 °C following 20 min. 75 °C incubation time and cooling down to 12 °C. Purified products were checked on agarose gel. To estimate the DNA concentration a mass marker (BioRad) and Nanodrop Spectrophotometer ND-1000 (peqLab) was used. Cycle sequencing reactions of the 28S Odonata were carried out using BigDye ReadyMix (Applied Biosystems). After standard ethanol-precipitation sequencing products were analyzed on an ABI 377 sequencer (Applied Biosystems). Cycle Sequencing reactions of apterygots, myriapods and crustaceans were performed using DNA Quick Start Mastermix (Beckman Coulter). CS products were ethanol-precipitated or purified with CleanSeq magnetic bead system (Agencourt) followed by sequencing on Beckman Coulter capillary sequencers CEQTM 8000 and CEQTM 8800.

28S rRNA PCR-products of apterygote hexapods and myriapods were purified loading samples on a 1% agarose gel (TBE buffer 1x). Bands were cut and purified using peqGOLD Gel Extraction Kit (peqLab Biotechnologie GmbH). Sequencing was carried out on ABI 3130xl Genetic Analyzer. Sequencing of the 28V/D10bPAUR fragment of Pauropodidae sp. required cloning. Purified PCR products were cloned into pCR 2.1-TOPO and chemically transformed into TOP10F' competent cells (2 μl TOPO Cloning reaction, 25 μl component *E. coli* cells). 15 μl of transformation product were spread on selective plates (500 μl ampicillin (50 μg/ml), 40 μl (40 mg/ml) X-gal, 40 μl (100 mM) IPTG and incubate over night. Twelve colonies were picked and after checking five of them were sequenced using vector primer (M13 Rv, M13 Fw, TOPO TA Cloning Kit, Invitrogen) on an ABI 3130xl Genetic Analyzer.

PCR-products (28S) of Pterygota were precipitated for purification (2 μl 4 M NHAc, 240 μl 98% ethanol, washing: 1 ml 70% ethanol, resuspended in 20 μl HPLC H_2O) for the primer combinations 28S rD1.2a/28S Rd4.2b, 28S rD3.2a/28S B and 28S A/28S Rd6.2b. PCR-Products amplified with 28ee/28hh were purified with MultiScreen PCR Plate (Millipore), purified products were sequenced at Macrogen (Korea). All PCR primers were used for sequencing also, 28mm was used as reverse sequencing primer. Fragments amplified with primer combinations 28ll/28jj, 28ll/28hh and 28w/28jj were cleaned with MultiScreen PCR Plate (Millipore) System. Cycle Sequencing was carried out using DYEnamic ET Dye Terminator Cycle Sequencing Kit (Amersham Bioscience). The sequencing reactions were purified again with the Montage SEQ Kit (Millipore) and sequenced on a MegaBACE 1000 system (Amersham Bioscience). 28y/28z fragments

were precipitated (see above). Using BigDye Terminator v3.1 Cycle Sequencing Kit (Applied Biosystems) cycle sequencing products were purified with Sephadex G-50 Superfine (GE Healthcare) and sequenced on a ABI PRISM 310 Genetic Analyzer (Applied Biosystems).

References

1. Giribet G, Ribera C: **A review of arthropod phylogeny: New data based on ribosomal DNA sequences and direct character optimization**. *Cladistics* 2000, **16**(2):204–231.

2. Chalwatzis N, Baur A, Stetzner E, Kinzelbach R, Zimmermann FK: **Strongly expanded 18S rRNA genes correlated with a peculiar morphology in the insect order of Strepsiptera**. *Zoology (Jena)* 1995, **98**:115–126.

3. Giribet G, Carranza S, Baguñà J, Riutort M, Ribera C: **First molecular evidence for the existence of a Tardigrada + Arthropoda clade**. *Mol Biol Evol* 1996, **13**:76–84.

4. Maddison DR, Baker MD, Ober KA: **Phylogeny of carabid beetles as inferred from 18S ribosomal DNA (Coleoptera: Carabidae)**. *Syst Entomol* 1999, **24**:103–138.

5. De Salle R, Gatesy J, Wheeler W, Grimaldi D: **DNA sequences from a fossil termite in Oligo-Miocene amber and their phylogenetic implications**. *Science* 1992, **257**(5078):1933–1936.

6. Dreyer H, Wägele JW: **Parasites of crustaceans (Isopoda: Bopyridae) evolved from fish parasites: molecular and morphological evidence**. *Zoology (Jena)* 2001, **103**:157–178.

7. Giribet G, Wheeler WC: **Phylogeny of the arachnid order Opiliones (Arthropoda) inferred from a combined approach of complete 18S and partial 28S ribosomal DNA sequences and morphology**. *Mol Phylogenet Evol* 1999, **11**(2):296–307.

8. Schlötterer C, Hauser MT, von Haeseler A, Tautz D: **Comparative evolutionary analysis of rDNA ITS regions in *Drosophila***. *Mol Biol Evol* 1994, **11**(3):513–522.

9. Nunn GB, Theissen BF, Christensen B, Arctander P: **Simplicity-correlated size growth of the nuclear 28S ribosomal RNA D3 expansion segment in the crustacean order Isopoda**. *J Mol Evol* 1996, **42**(2):211–223.

10. Hillis DM, Dixon MT: **Ribosomal DNA: molecular evolution and phylogenetic inference**. *The Quarterly Review of Biology* 1991, **66**(4):411–453.

11. Dell'Ampio E: **Il gene codificante per l'rRNA 28S: evoluzione della struttura secondaria ed utilità come marcatore filogenetico in alcune specie della famiglia Neanuridae (Hexapoda, Collembola)**. *PhD thesis*, University of Siena, ItalyDepartment of Evolutionary Biology 2003.

12. Whiting MF: **Mecoptera is paraphyletic: multiple genes and phylogeny of Mecoptera and Siphonaptera Mecoptera is paraphyletic: multiple genes and phylogeny of Mecoptera and Siphonaptera**. *Zool Sci* 2002, **31**:93–104.

13. Kück P: **Phylogenetic Reconstruction of relationships in the suprafamily Aeshnoidea (Anisoptera). Based on the analysis of 28S rRNA and its secondary structure**. *Master's thesis*, Zoologisches Forschungsmuseum A. Koenig, Molecular Lab, University of Bonn, Germany 2006.

14. Schmidt C: **Phylogeny of Libellulidae (Insecta: Odonata) based on molecular analysis of the complete nuclear 28S rRNA gene including combined nucleotid (DNA)/doubled (RNA) substitution models**. *Master's thesis*, University of Bonn, Germany, Zoologisches Forschungsmuseum A. Koenig, Molecular Lab 2006.

15. Letsch HO: **Phylogeny of Anisoptera (Insecta: Odonata): Promises and limitations of a new alignment approach**. *PhD thesis*, University of Bonn, Germany, Zoologisches Forschungsmuseum A. Koenig, Molecular Lab 2007.

16. Dell'Ampio E, Carapelli A, Frati F: **Secondary structure and sequence variation of the 28S rRNA gene in the Neanuridae, and its utility as a phylogenetic marker: Proceedings of the Xth international Colloquium on Apterygota, české Budějovice 2000: Apterygota at the Beginning of the Third Millennium**. *Pedobiologia (Jena)* 2002, **46**(3-4):274–283.

17. Van der Auwera G, Chapelle S, De Wachter R: **Structure of the large ribosomal subunit RNA of *Phytophthora megasperma*, and phylogeny of the oomycetes**. *FEBS Lett* 1994, **338**(2):133–136.

18. Mallatt J, Sullivan J: **28S and 18S rDNA sequences support the monophyly of lampreys and hagfishes**. *Mol Biol Evol* 1998, **15**(12):1706–1718.

19. Chou Q, Russell M, Birch DE, Raymond J, Bloch W: **Prevention of pre-PCR mis-priming and primer dimerization improves low-copy-NUMBER amplifications**. *Nucleic Acids Res* 1992, **20**(7):1717–1723.
